# Indications for Cocaine in Surgical Operations

**Published:** 1894-08

**Authors:** 


### SELECTIONS.

### Indications for Cocaine in Surgical Operations.

Prof. A. Ceci (*Gazzetta Degli Ospitali*, No. 29, 1894), has employed cocaine in 484 operations since 1885, using it in cases where general anæsthesia is usually practiced—for example, in eight operations for extirpation of goitre, in two extirpations of the mammary gland with cleaning out of the axilla, thirty-four radical operations for hernia, two vaginal hysterectomies with removal of the uterine appendages, one laparotomy, four amputations of the tongue, two of which were preceded by preliminary ligation of the linguals, with cleaning out of the submaxillary region. First he employed such strong solutions as five to ten per cent.; later he only used from a one to one-half per cent. solution, which latter he now exclusively employs. The solutions must be recent and contain two and one-half per cent. of boric acid. In adults he gives a preliminary injection of morphine, so that the anæsthesia is mixed (morphio-cocaine). During the first two years he only observed two cases of intolerance of the drug, which were due to too strong solutions being used. The fluid should be well scattered throughout the tissues, and care be taken that it is not injected into a vein. He denies that cocaine predisposes to suppuration of the part; this is dependent either upon lack of antisepsis, pollution of the solution, or a dirty syringe. During the past year he was able to reduce the number of chloroformizations in his clinic to twenty-three, while the year before they amounted to seventy. He finds cocaine of especial value in operations on the neck, in extirpations of goitres and lymphatic glands, and in radical operations for hernia. Contrary to the opinions generally expressed he has found that cocaine anæsthesia may last over an hour without repetition of the injection. *The Cincinnati Lancet Clinic.*

